# Identification of an EMT-related lncRNA signature and LINC01116 as an immune-related oncogene in hepatocellular carcinoma

**DOI:** 10.18632/aging.203888

**Published:** 2022-02-11

**Authors:** Haisu Tao, Yuxin Zhang, Tong Yuan, Jiang Li, Junjie Liu, Yixiao Xiong, Jinghan Zhu, Zhiyong Huang, Ping Wang, Huifang Liang, Erlei Zhang

**Affiliations:** 1 Hepatic Surgery Center, Tongji Medical College, Tongji Hospital, Huazhong University of Science and Technology, Wuhan, China; 2 Hubei Key Laboratory of Hepato-Pancreato-Biliary Diseases, Wuhan, China; 3 Department of Hepatobiliary Surgery, The First Affiliated Hospital of Guangzhou Medical University, Guangzhou, China

**Keywords:** HCC, EMT, prognostic signature, LINC01116, immune

## Abstract

Background: Epithelial–mesenchymal transition (EMT) plays a critical role in the recurrence and metastasis of hepatocellular carcinoma (HCC). Some long noncoding (lnc)RNAs are involved in this process through the regulation of EMT-related transcription factors.

Methods: In this study, we established a novel EMT-related lncRNA signature in HCC and identified hub lncRNAs that can serve as potential therapeutic targets. Differentially expressed lncRNAs were identified by screening HCC patient data from The Cancer Genome Atlas, and a correlation analysis was performed to identify those associated with EMT. The EMT-related lncRNA signature was established by univariate, least absolute shrinkage and selection operator, and multivariate Cox regression analyses. After verifying the prognostic accuracy of the signature, its relationships to immune cell infiltration and immune checkpoint targets were explored. LINC01116 was identified as a hub lncRNA and its role in HCC was investigated *in vitro* and *in vivo*.

Results: A 5-lncRNA signature was developed for HCC and its prognostic accuracy was assessed by survival, time-dependent receiver operating characteristic curve, clinical correlation, and Cox regression analyses. The correlation analysis showed that the lncRNA signature was closely related to immune cell infiltration and 10 immune checkpoint targets and also predicted the prognosis of HCC patients with high accuracy. *In vitro* and *in vivo* experiments revealed that LINC01116 stimulated cell proliferation, cell cycle progression, and tumor metastasis. We also found that LINC01116 was closely related to immune regulation.

Conclusions: These results demonstrate that LINC01116 is an immune-related oncogene that is associated with both EMT and immune regulation in HCC. Moreover, the EMT-related lncRNA signature that includes LINC01116 can guide risk stratification and clinical decision-making in HCC management.

## INTRODUCTION

Liver cancer is among the most deadly tumors and the second leading cause of cancer-related mortality worldwide [1]. Hepatocellular carcinoma (HCC), the most common type of liver cancer, is often diagnosed at relatively advanced stages, such that patients miss the optimal time window for surgery [2]. Even after surgical resection and standardized treatment, the incidence of recurrence and distant metastasis in HCC remain high [3]. At advanced stages, there are few treatment options and the prognosis is poor. Clarifying the mechanism of HCC progression can identify novel diagnostic and prognostic biomarkers for improved clinical outcomes.

The regulation of epithelial–mesenchymal transition (EMT), a hallmark of cancer, is an important mechanism underlying HCC metastasis [4–6]. Tumor epithelial cells lose contact with each other and gain motility as a result of the dissolution of cell–cell junctions formed by E-cadherin, and spread to adjacent and distant tissues [7]. Transforming growth factor (TGF)-β is involved in both fibrogenesis and carcinogenesis through the regulation of EMT [7]. EMT markers such as E-cadherin, β-catenin, and mucin (MUC)15 related to vascular invasion and poor differentiation are aberrantly expressed in HCC patients [8] and are promising markers for HCC diagnosis and treatment.

Abnormal expression of long noncoding (lnc)RNAs has been observed in multiple types of cancer. Some lncRNAs participate in EMT through interaction with transcriptional regulators. For example, the lncRNA homeobox antisense intergenic RNA (HOTAIR) modulates the interaction between SNAIL and enhancer of zeste homolog (EZH)2 via a SNAIL/HOTAIR/EZH2 repressor complex that promotes EMT [9], while the lncRNA metastasis-associated lung adenocarcinoma transcript (MALAT)1 acts as an oncogene by interacting with EZH2 or functioning as a competing endogenous (ce)RNA for several members of the miR-200 micro (mi)RNA family that negatively regulate EMT-related factors [10]. Additionally, the lncRNA H19 regulates multiple genes involved in EMT progression by acting as a ceRNA [11].

EMT-associated gene models have been developed to predict HCC prognosis [4, 12]. However, the functions and prognostic value of EMT-related lncRNAs have not been systematically investigated. In this study, we developed an EMT-related lncRNA signature for predicting HCC prognosis using data from The Cancer Genome Atlas (TCGA) database. We validated the prognostic value of the signature and investigated its relationship with immune cell infiltration- and immune checkpoint-related factors. Finally, we explored the role of 5 hub lncRNAs in HCC and the function of one of these (LINC01116) *in vitro* and *in vivo*. Our results demonstrate for the first time that LINC01116 acts as an immune-related oncogene in HCC and has clinical utility as a prognostic biomarker.

## MATERIALS AND METHODS

### Data collection and processing

Gene expression data of 424 samples including 374 tumor and 50 normal samples and clinical information of 374 HCC patients were downloaded from TCGA database. LncRNAs and mRNAs were identified from RNA sequence data based on annotations in the human GENCODE database. The “edgeR” package in R software was used to identify differentially expressed lncRNAs with the threshold P value <0.05 and |log(fold change)|>2. Additionally, the data of 368 HCC patients with complete clinical information were randomly divided into training and validation sets at a 1:1 ratio. The clinical characteristics of the 2 groups were similar.

### EMT-related lncRNA identification

EMT-related genes were obtained from the HALLMARK_EPITHELIAL_MESENCHYMAL_TRANSITION gene set in Molecular Signatures Database v7.4 (https://www.gsea-msigdb.org/gsea/msigdb/). EMT-related lncRNAs were identified by correlation analysis based on a P value <0.001 and absolute Pearson coefficient >0.4.

### Construction of the EMT-related lncRNA signature

**Figure 1:**
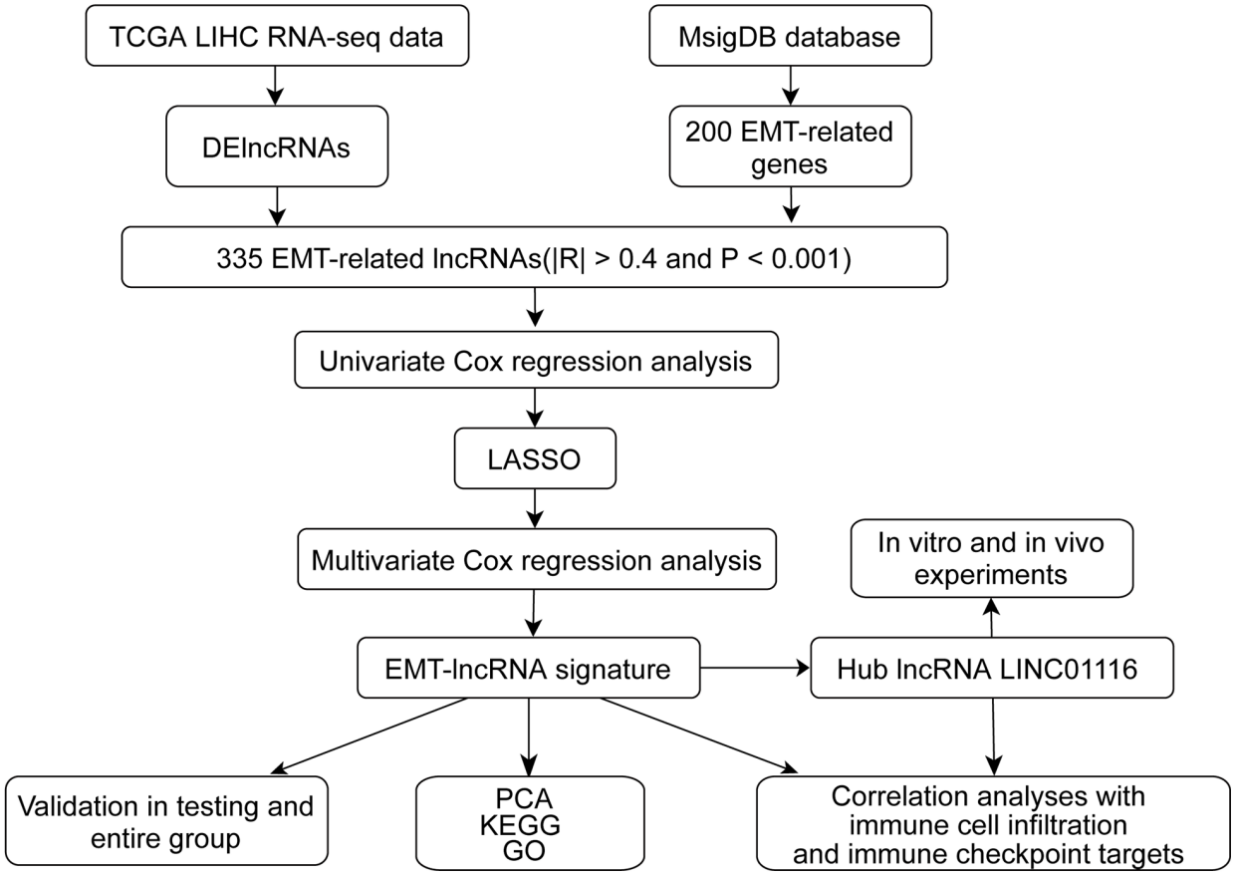
The flow chart of this study.

A flow diagram of the study is shown in Figure 1. In order to establish an EMT-related lncRNA signature for predicting prognosis in HCC, we carried out a univariate Cox regression analysis to screen prognostic lncRNAs (P<0.05), followed by least absolute shrinkage and selection operator (LASSO) penalized Cox regression and multivariate Cox regression analyses to select those that were relevant. The lncRNA expression level and regression coefficient β from the multivariate Cox regression results were used to develop the following equation: risk score=β lncRNA1×lncRNA1 expression+β lncRNA2×lncRNA2 expression+/+β lncRNA n×lncRNA n expression. After assessing the prognostic value of the signature, the “Survminer” package in R was used to determine the optimal cutoff value for dividing patients into high- and low-risk groups.

### Validation of the prognostic significance of EMT-related lncRNAs

The prognostic value of the EMT-related lncRNA signature was verified by Kaplan–Meier and receiver operating characteristic (ROC) curve analyses in the training and validation sets and in the combined dataset (i.e., the whole patient population). The relationship between risk score and survival is presented as a risk score distribution map and lncRNA expression heatmap. Combined with other clinical characteristics of HCC patients, uni- and multivariate Cox regression analyses were carried out to determine whether the risk score was an independent prognostic factor in HCC. The prognostic accuracy of the signature and other clinical features in all groups was verified by ROC curve analysis.

### Analysis of the relationship between the prognostic signature, immune cell infiltration, and immune checkpoint targets

The CIBERSORT algorithm was used to analyze the abundance of 22 types of tumor-infiltrating immune cell (TIIC) and Tumor Immune Estimation Resource (TIMER) was used to calculate the abundance of 6 different types of TIIC. The association between the lncRNA signature and the different types of TIIC was analyzed to determine whether the signature can predict immune cell infiltration in HCC.

The success of programmed death (PD)-1/programmed death ligand (PD-L)1 and cytotoxic T-lymphocyte antigen (CTLA)-4 blockade for HCC treatment has intensified research efforts to identify novel immune checkpoint targets. In this study, we examined established targets as well as those with therapeutic potential such as T-cell immunoglobulin and mucin domain-containing (TIM)-3, indoleamine 2,3-dioxygenase (IDO)1, glucocorticoid-induced tumor necrosis factor receptor-related (GITR), histone deacetylase (HDAC)2, B7-histone (H)3, and V-domain Ig suppressor of T cell activation (VISTA) [13–15]. In order to evaluate the role of the lncRNA signature in the treatment response to immune checkpoint blockade (ICB), we performed a correlation analysis between the signature and the expression level of 10 immune checkpoint targets in HCC.

### Tissue specimens and cell culture

We collected 50 paired HCC tissue and adjacent nontumor tissue samples from patients who underwent hepatectomy between March 2016 and October 2017 at the Hepatic Surgery Center of Tongji Hospital. OS information was collected through electronic medical records or telephone follow-up. Informed consent forms to donate their tissue samples for biomedical research, which were approved by the ethics committee of Tongji Hospital, were signed by all patients. Huh7 and SK-Hep1 HCC cell lines were purchased from the Liver Cancer Institute of Fudan University (Shanghai, China). The cells were cultured in Dulbecco’s Modified Eagle’s Medium supplemented with 10% fetal bovine serum at 37° C and 5% CO_2_.

### Immunofluorescence analysis

HCC cells were cultured on coverslips in 24-well plates for 24 h. After 3 washes with phosphate-buffered saline, the cells were fixed with 4% paraformaldehyde for 15 min and permeabilized with 0.1% Triton X-100 for 10 min. After culturing in medium containing 1% bovine serum albumin for 2 h, the cells were incubated overnight at 4° C with rabbit anti–E-cadherin and mouse anti-vimentin antibodies and then labeled with Alexa Fluor 568- or 488-conjugated secondary antibody (from different species) for 2 h. Cell nuclei were stained with 4′, 6-diamidino-2-phenylindole for 10 min. The coverslips were mounted and observed with a confocal microscope.

### Fluorescence *in situ* hybridization (FISH)

Cells were cultured on coverslips in 24-well plates. LINC01116 was detected in HCC cells by FISH using a probe and commercial kit from RiboBio (Guangzhou, China) according to the manufacturer’s instructions. The labeled cells were observed with a confocal microscope.

### Xenograft tumor model

Male BALB/c nude mice (4 weeks old, weighing 15 g) were maintained under specific pathogen-free conditions. Huh7 cells transfected with empty vector or LINC01116 overexpression plasmid (3×10^6^) were subcutaneously injected into the back of the mice on the right and left sides, respectively. Tumor growth was observed every 3 days and tumors were harvested after 3 weeks.

### Immunohistochemistry

Tumor tissues from mice were fixed in 4% paraformaldehyde solution and then dehydrated in ethanol solutions, embedded in paraffin, and cut into sections that were incubated overnight at 4° C with primary antibodies against Ki67, vimentin, and E-cadherin followed by horseradish peroxidase-conjugated secondary antibody for 1 h at room temperature. Images of representative fields were acquired with a C1 Digital ECLIPSE microscope.

### Cell proliferation and migration assays and cell cycle analysis

The cell proliferation (Cell Counting Kit [CCK]-8), 5-ethynyl-2′-deoxyuridine (EdU), transwell migration, and wound healing assays and cell cycle analysis were carried out as previously described [16].

### Statistics analysis

Data analysis were conducted using R software (version 3.6.3) and Prism 7.0 software. Data are presented as mean ± SD. Qualitative variables were analyzed by Student's t test, one-way ANOVA and Pearson correlation test were used to analyze qualitative variables. P<0.05 was considered to be statistically significant.

### Data availability statement

Publicly available datasets were analyzed in this study. The data are accessible at the following repositories: https://portal.gdc.cancer.gov/repository.

### Ethics statement

This research protocol was approved by the ethical committee of Tongji Hospital and a consent form was signed by each participating patient.

## RESULTS

### Identification of EMT-related lncRNAs

**Figure 2:**
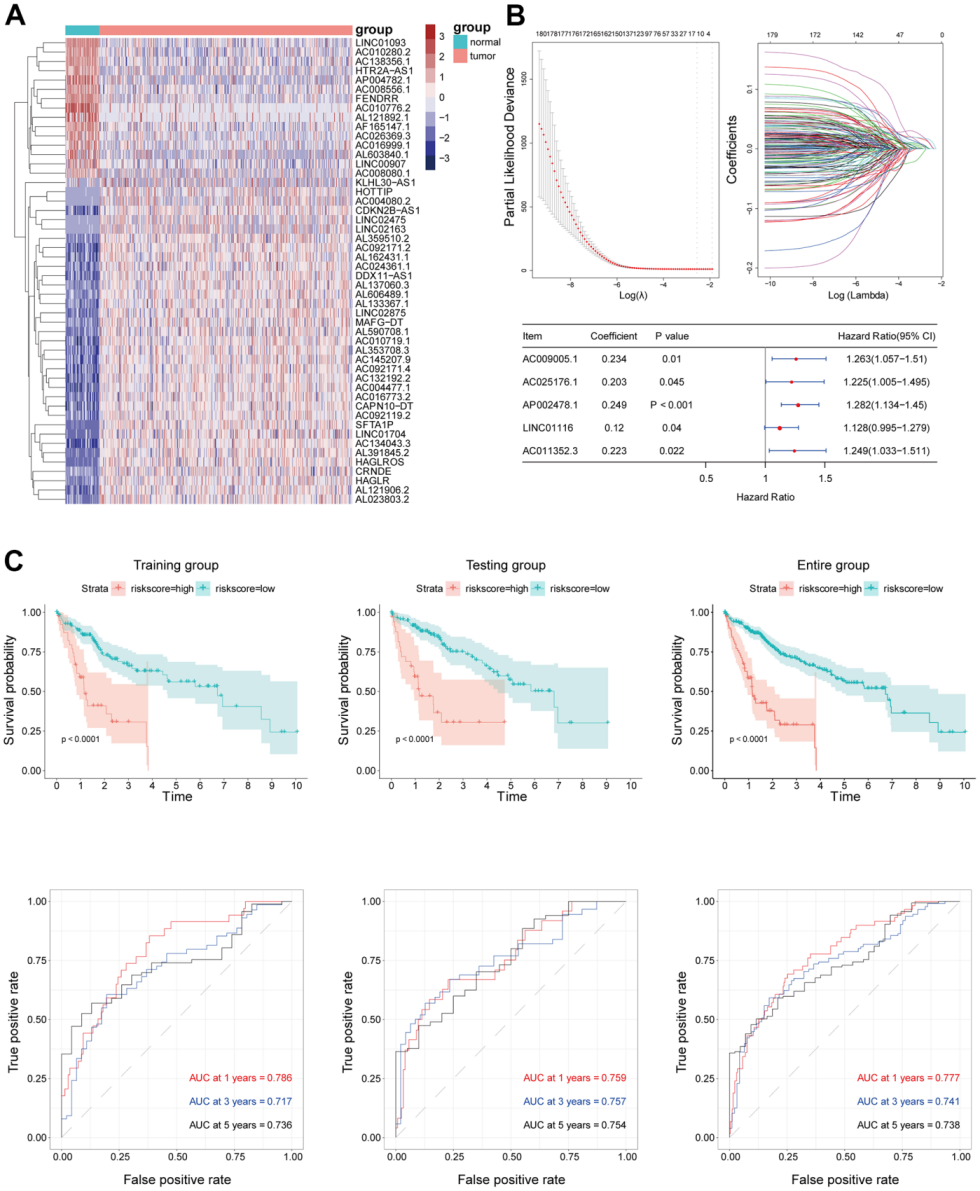
(**A**) The top 50 of both up- and down-regulated DElncRNAs with the most significant differences were represented by the heatmap. (**B**) EMT-related lncRNA signature was established by Univariate Cox regression, LASSO and Multivariate Cox regression analysis. (**C**) Kaplan–Meier survival analysis suggested a lower OS in high-risk groups. ROC curve showed good accuracy of this signature in predicting OS of 1, 3 and 5 years.

Using a cutoff of |log_2_(fold change)|>2 and P<0.05, we identified 1283 differentially expressed lncRNAs (84 downregulated and 1199 upregulated) in 374 HCC samples compared to 50 normal samples. The top 50 lncRNAs were used to construct a heatmap (Figure 2A). We also identified 200 EMT-related genes from the Molecular Signatures Database; by correlation analysis, 335 EMT-related lncRNAs were identified based on a threshold of P<0.001 and absolute Pearson coefficient >0.4.

### Construction of the EMT-related lncRNA signature in HCC

A total of 368 TCGA HCC patients with complete clinical information were randomly assigned to the training (n=197) and validation (n=171) sets. In order to identify lncRNAs with prognostic value in HCC, the EMT-related lncRNAs were evaluated by univariate Cox analysis; 180 were found to be significantly related to the overall survival (OS) of HCC patients. LASSO and multivariate Cox regression analyses were performed to select and validate the prognostic lncRNAs in the training cohort (Figure 2B). An EMT-related prognostic lncRNA signature for HCC was established based on the regression coefficients and expression levels of these lncRNAs that included AC011352.3, AC009005.1, AC025176.1, AP002478.1, and LINC01116.

The Kaplan–Meier analysis of the training and validation sets and whole patient population indicated that HCC patients with a high risk score had a shorter OS than those with a low risk score. The ROC curve analysis for the prognostic lncRNA signature showed excellent predictive accuracy, with an area under the ROC curve (AUC) of 0.786, 0.717, and 0.736 for predicting 1-, 3-, and 5-year OS, respectively. The prognostic value of the signature was assessed in the validation set and whole patient population; the AUCs for predicting 1-, 3-, and 5-year OS were 0.759, 0.757, and 0.754, respectively, in the validation set and 0.777, 0.741, and 0.738, respectively, in the whole population (Figure 2C).

### Validation of the prognostic lncRNA signature

**Figure 3:**
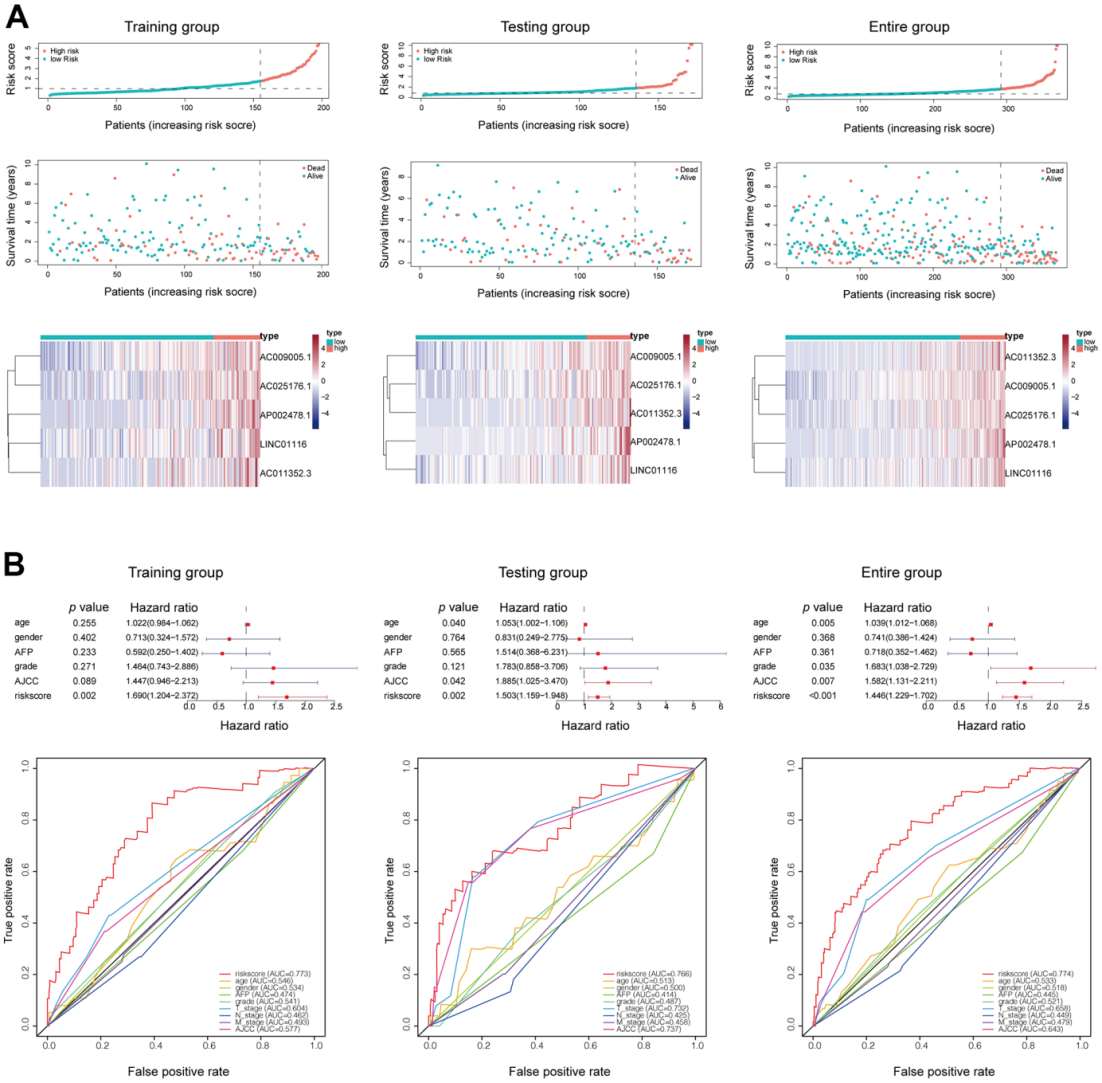
(**A**) Mortality status and lncRNAs expression in each patient were plotted according to the ordered risk score. (**B**) Multivariate and ROC curves confirmed the EMT-related lncRNA signature as an independent prognostic factor for HCC.

The mortality rate in all groups increased with risk score; meanwhile, the expression levels of the 5 hub lncRNAs in the EMT-related signature also increased (Figure 3A). A multivariate Cox regression analysis that also included other clinical characteristics of HCC patients showed that risk score was an independent prognostic factor in HCC, with a hazard ratio of 1.690 (1.204−2.372) in the training set, 1.503 (1.159−1.948) in the validation set, and 1.446 (1.229−1.702) in the whole patient population (Figure 3B). The risk score had a higher AUC than the other clinical features in each group, confirming its value as an accurate prognostic factor (Figure 3B).

**Figure 4:**
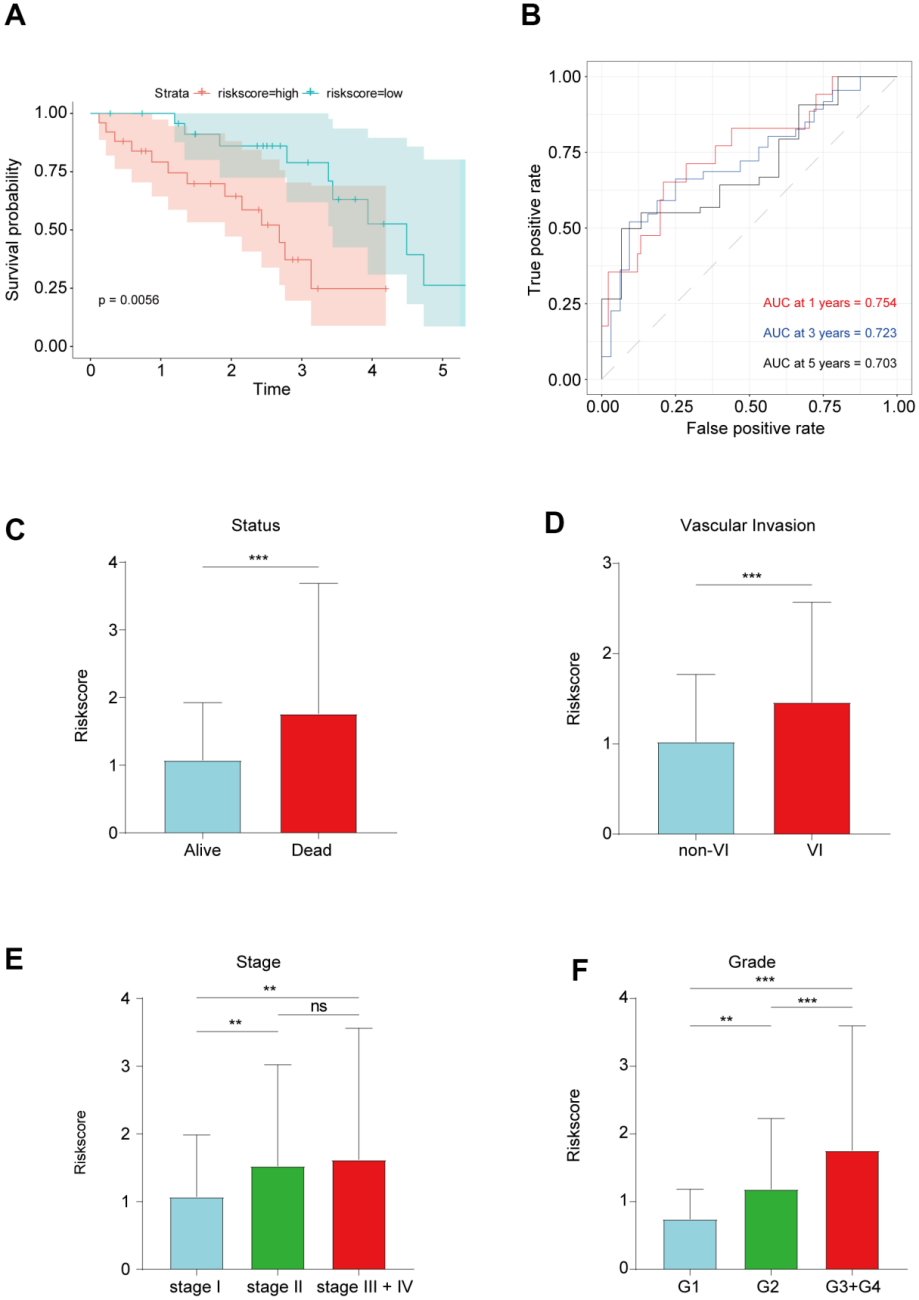
(**A**) Kaplan–Meier survival analysis in Tongji cohort. (**B**) ROC curve showed good accuracy of this signature in Tongji cohort. (**C**) The association between risk score and living status. (**D**) The association between risk score and vascular invasion. (**E**) The association between risk score and tumor grade. (**F**) The association between risk score and tumor stage. **P < 0.01 and ***P < 0.001.

To investigate the robustness of the prognostic lncRNA signature, we evaluated its predictive accuracy in the Tongji cohort including 50 HCC patients. Consistent with the results from the TCGA cohort, that HCC patients with a high risk score had a shorter OS than those with a low risk score (Figure 4A). In addition, the prognostic lncRNA signature had an AUC of 0.754 at 1 year, 0.723 at 3 years, and 0.703 at 5 years in the Tongji cohort (Figure 4B), indicating an excellent predictive accuracy. Then, we analyzed the relationships between the EMT-related lncRNA signature and clinicopathologic characteristics of HCC patients. The risk score was significantly higher in patients with vascular invasion and those who died (Figure 4C, 4D). We also observed that the risk score tended to increase with tumor grade and stage (Figure 4E, 4F), implying that the signature reflects the high malignancy of HCC.

### Functional analysis of the prognostic signature

**Figure 5:**
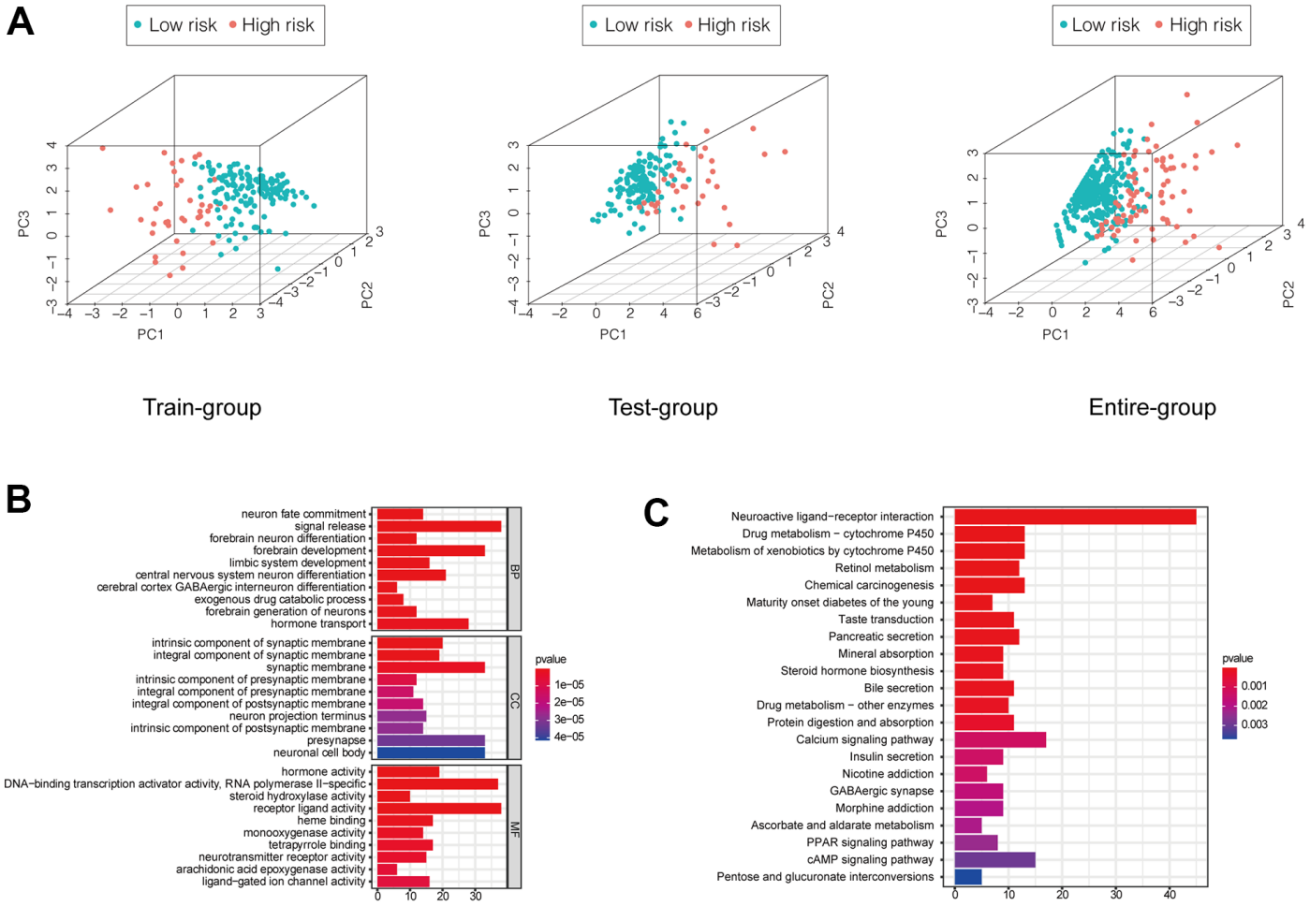
(**A**) PCA showed a scattered distribution of patients in each group. (**B**, **C**) GO and KEGG analysis of DEGs between high and low-risk groups of the entire group.

We performed a principal component analysis to compare low and high-risk patients in the training and validation sets and the whole patient population who were classified based on the EMT-related lncRNA signature. The low- and high-risk groups formed distinct clusters, confirming the prognostic accuracy of the signature (Figure 5A). We also explored the function of lncRNAs in the signature by performing a correlation analysis with mRNAs that were differentially expressed between high- and low-risk groups, whose functions were predicted by Gene Ontology and Kyoto Encyclopedia of Genes and Genomes pathway analyses. It showed that function annotations of this signature was enriched in terms related to signal release, intrinsic component of synaptic membrane, hormone activity and neuroactive ligand−receptor interaction (Figure 5B, 5C).

### Correlation between the prognostic signature, immune cell infiltration, and immune checkpoint targets

**Figure 6:**
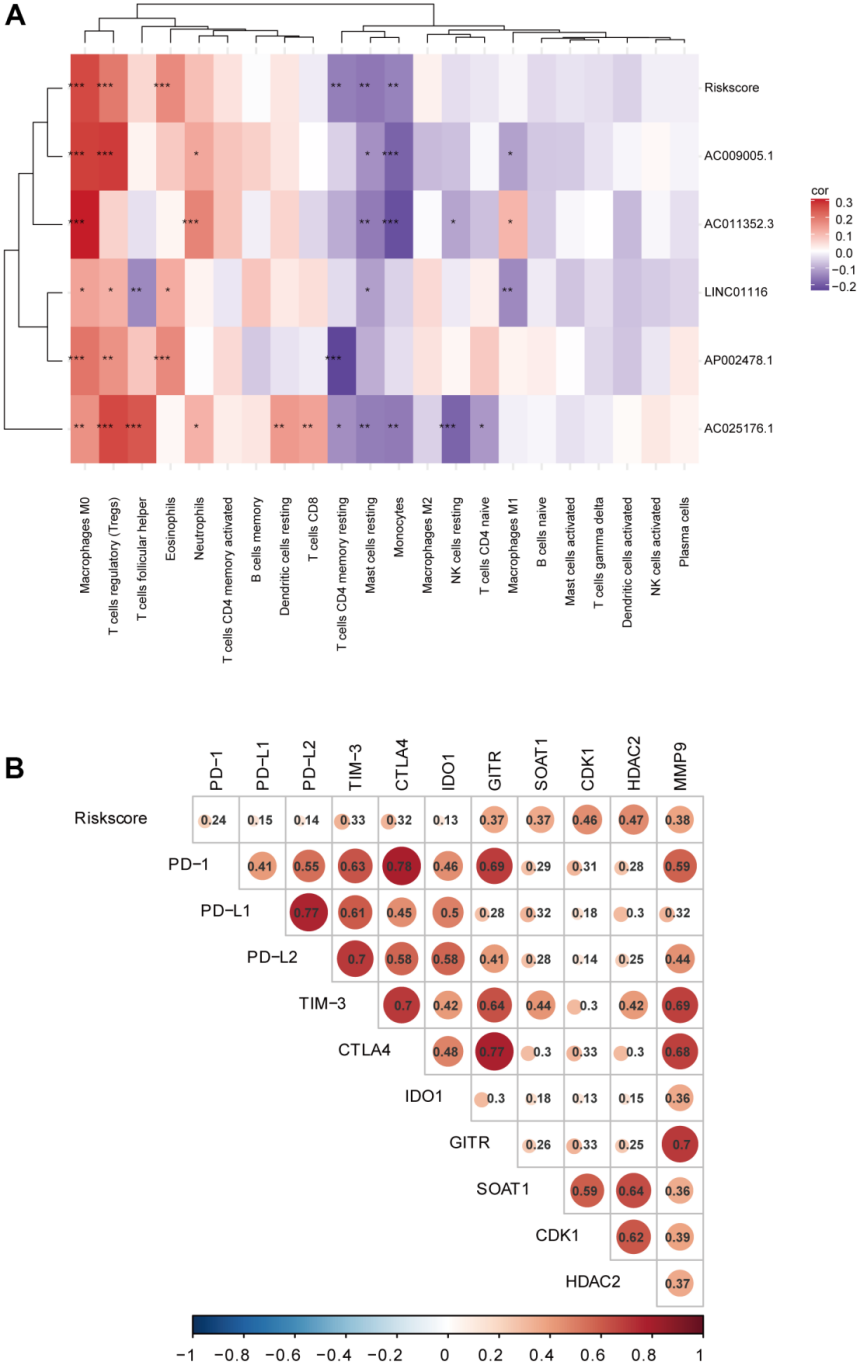
(**A**) Correlation analyses of the EMT-related lncRNA signature with immune cell infiltration. (**B**) Correlation analyses of the EMT-related lncRNA signature with immune checkpoint targets.

Using the CIBERSORT algorithm, we investigated whether the EMT-related lncRNA signature was related to immune cell infiltration in HCC. The results of the correlation analysis revealed a significant correlation with infiltrating resting memory cluster of differentiation (CD)4+ T cells (r=−0.154; p=3.08e−03), regulatory T cells (r=0.197; p=1.46e−04), monocytes (r=-0.149; p=4.27e−03), macrophages M0 (r=0.265; p=2.53e−07), resting mast cells (r=−0.163; p=1.69e−03), and eosinophils (r=0.171; p=9.73e−04; Figure 6A). Additionally, all lncRNAs in the signature showed a significant correlation with the infiltration of multiple immune cell types.

To investigate the potential role of the EMT-related lncRNA signature in ICB therapy for HCC, we performed a correlation analysis between risk score and 10 immune checkpoint targets (CTLA4, TIM-3, PD-L1, PD-L2, PD-1, IDO1, GITR, HDAC2, B7-H3, and VISTA) and found close correlations (Figure 6B), suggesting that the signature can predict the outcome of ICB therapy in HCC patients.

### Hub lncRNAs in HCC

**Figure 7:**
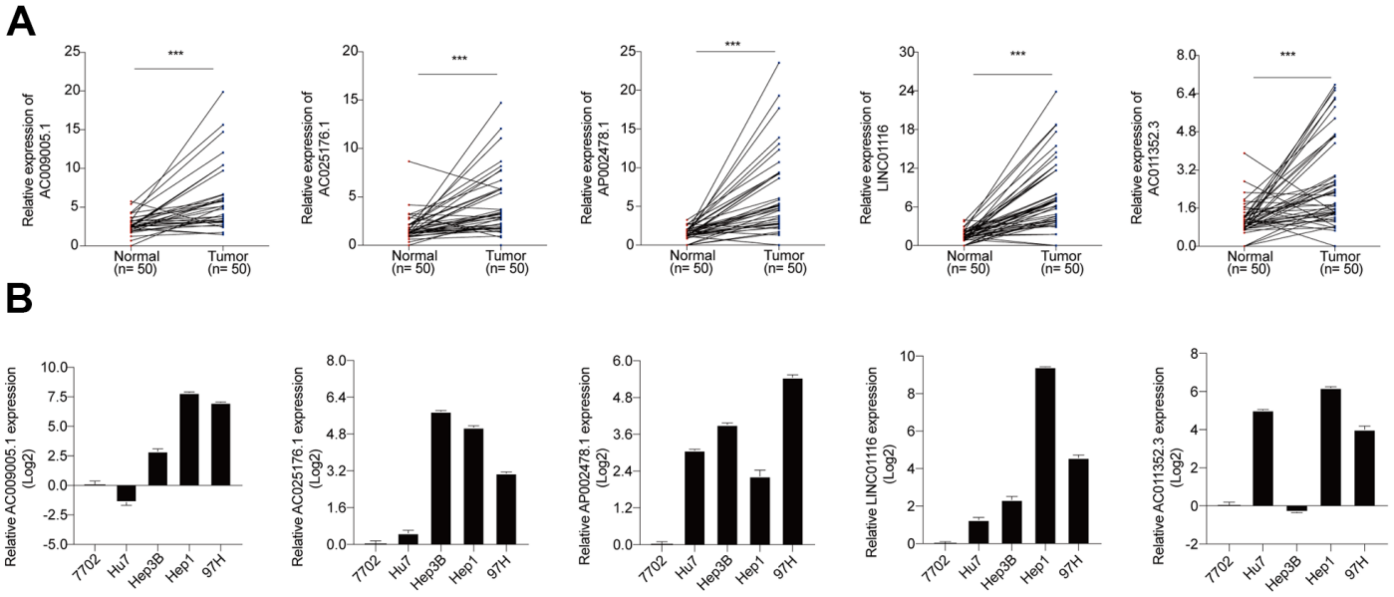
(**A**) The expression levels of 5 hub lncRNAs in HCC specimens of Tongji hospital. (**B**) The expression levels of 5 hub lncRNAs in normal liver cell line and 4 HCC cell lines.

Using TCGA data, the expression levels of the 5 hub lncRNA s in this signature were compared in the HCC tissues and normal liver tissues. It showed that all the 5 hub lncRNAs expression levels were higher in HCC tissues than these in the normal liver tissues. Besides, the Kaplan–Meier analysis indicated that the increased the 5 hub lncRNAs were significantly correlated with OS of HCC patients (Supplementary Figure 1). The increased levels of the 5 hub lncRNAs in HCC was confirmed in HCC specimens from the Tongji Hospital cohort, and the Kaplan–Meier analysis with the log-rank test indicated a significant correlation with OS (Figure 7A). Additionally, 3 of the lncRNAs (AC025176.1, LINC01116, and AC011352.3) were highly expressed in 4 HCC cell lines compared to a normal liver cell line (Figure 7B). Of these, LINC01116 was selected for further analysis based on the fact that it had the highest relative expression in clinical HCC specimens and has not been previously characterized in HCC.

### LINC01116 enhances HCC cell proliferation and regulates the cell cycle

**Figure 8:**
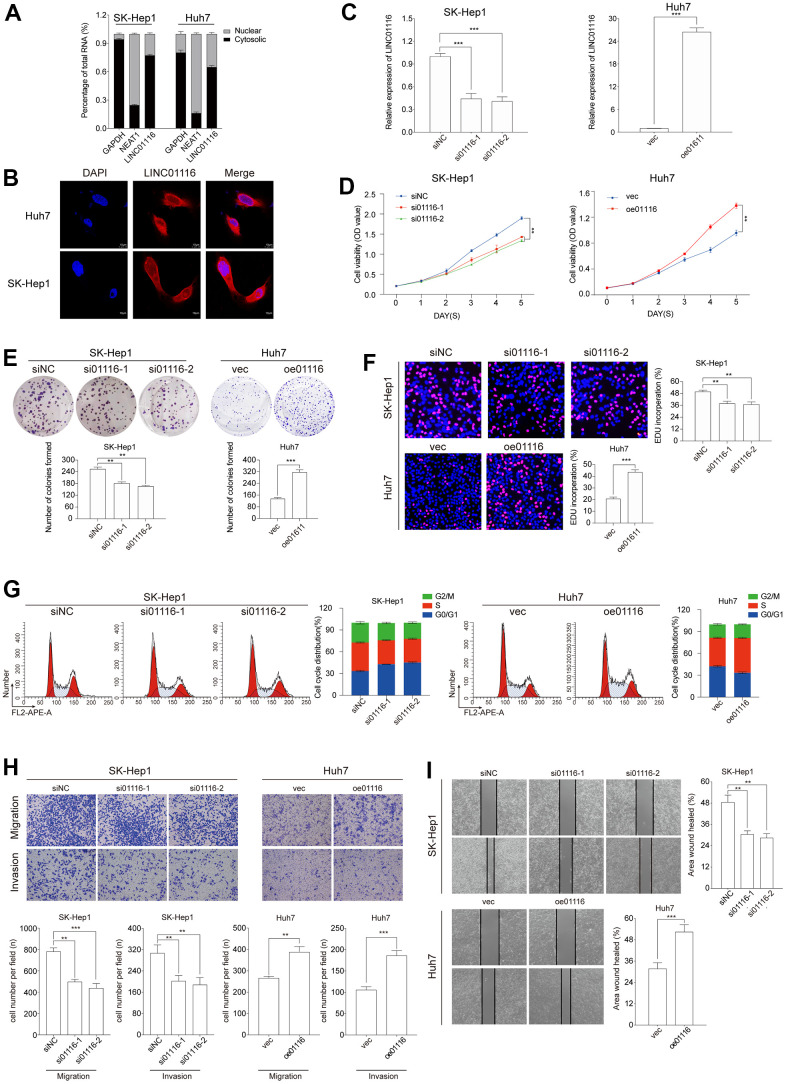
(**A**) The expression level of LINC01116 in the subcellular fractions of HCC cells was detected by qRT-PCR. (**B**) FISH assay analysis for the location of LINC01116 in HCC cells. (**C**) Transfection efficiency was verified by qRT-PCR. (**D**–**F**) Cell viability was evaluated with CCK-8, EdU and colony formation assays in HCC cells. (**G**) Cell cycle was examined by the flow cytometry. (**H**) Transwell assays were used to detect HCC cells invasion and migration. (**I**) Migration ability was evaluated by wound healing assay. **P < 0.01 and ***P < 0.001.

To determine the subcellular localization of LINC01116 in HCC cells, we performed cytoplasmic/nuclear fractionation and FISH in SK-Hep1 and Huh7 cells. LINC01116 was detected at a higher level in the cytoplasm than in the nucleus, implying that it mainly acts on cytoplasmic factors (Figure 8A, 8B).

To investigate the function of LINC01116 in HCC, we used 2 different small interfering (si)RNAs to silence its expression in SK-Hep1 cells. Conversely, LINC01116 expression was markedly increased in Huh7 cells transfected with LINC01116 overexpression plasmid (Figure 8C).

The effect of LINC01116 loss or gain of function on HCC cell proliferation and viability was evaluated with the CCK-8, EdU, and colony formation assays. The results showed that LINC01116 knockdown suppressed the proliferation of SK-Hep1 cells while LINC01116 overexpression enhanced Huh7 cell proliferation (Figure 8D–8F). We also examined cell cycle distribution under the 2 conditions by flow cytometry. LINC01116 silencing increased the proportion of SK-Hep1 cells arrested in G0/G1 phase whereas LINC01116 overexpression induced cell cycle progression in Huh7 cells (Figure 8G). In contrast, HCC cell apoptosis was unaffected by LINC01116 depletion and overexpression (Supplementary Figure 2).

### LINC01116 promotes EMT and metastasis in HCC

**Figure 9:**
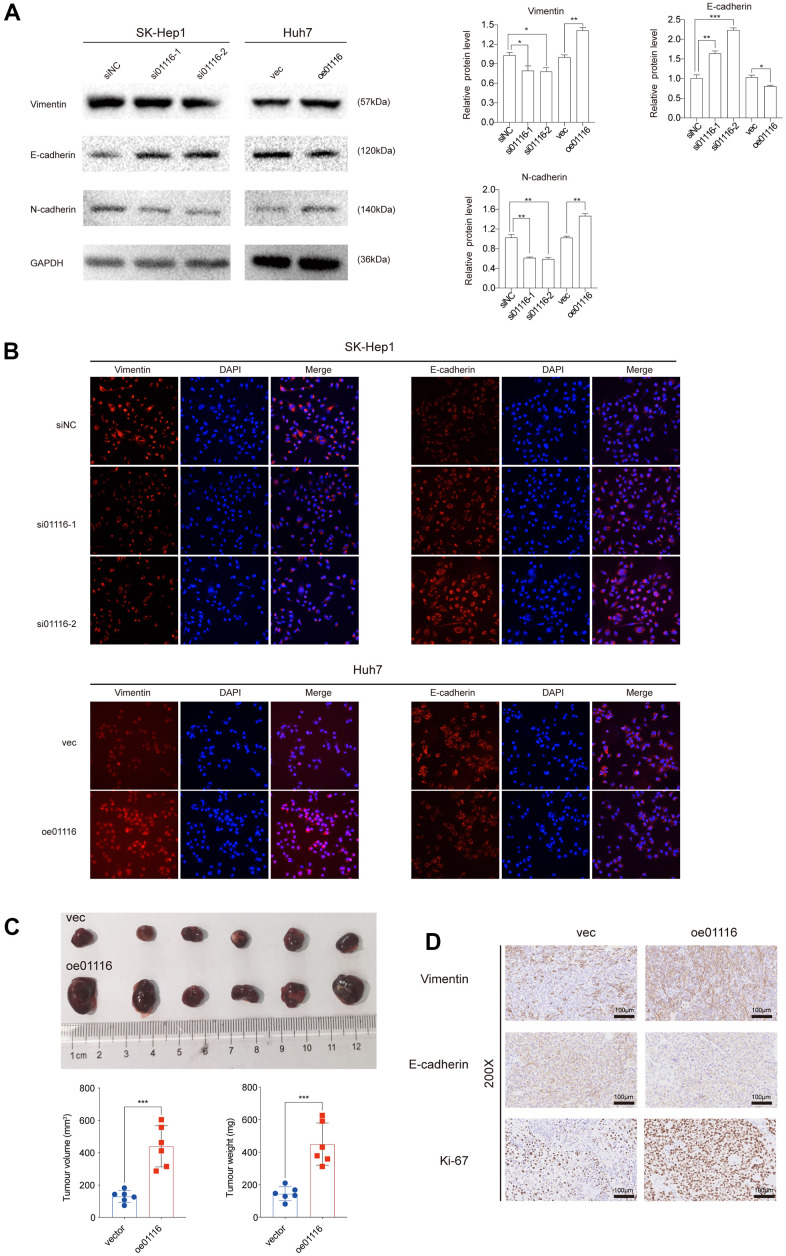
(**A**) EMT markers were examined via western blot analysis. (**B**) Immunofluorescence assay was used to detect EMT markers. (**C**) Image of subcutaneous tumor tissues. The volume and weight of tumors were measured. (**D**) Ki67, vimentin and E-cadherin were observed in subcutaneous tumor tissues by IHC. **P < 0.01 and ***P < 0.001.

To investigate the role of LINC01116 in the migration and invasion of HCC cells, we performed transwell migration and wound healing assays. LINC01116 knockdown suppressed migration and invasion in SK-Hep1 cells, while its overexpression had the opposite effect in Huh7 cells (Figure 8H). In the wound-healing assay, migration distance was decreased in SK-Hep1 cells transfected with siRNAs and was increased in Huh7 cells overexpressing LINC01116 (Figure 8I). We also examined the expression of the EMT markers vimentin, E-cadherin, and N-cadherin by western blotting and immunofluorescence analysis. LINC01116 knockdown in SK-Hep1 cells decreased the levels of vimentin and N-cadherin and increased that of E-cadherin, with LINC01116 overexpression have the opposite effects in Huh7 cells (Figure 9A, 9B).

To verify the above results *in vivo* we used a mouse xenograft model. Compared to the control group, Huh7 cells overexpressing LINC01116 formed larger tumors (Figure 9C); moreover, these tumors showed upregulation of Ki67 and vimentin and downregulation of E-cadherin by immunohistochemistry (Figure 9D). Collectively, these results indicate that LINC01116 promotes EMT and metastasis in HCC.

### LINC01116 is associated with immune cell infiltration and expression of immune checkpoint targets

**Figure 10:**
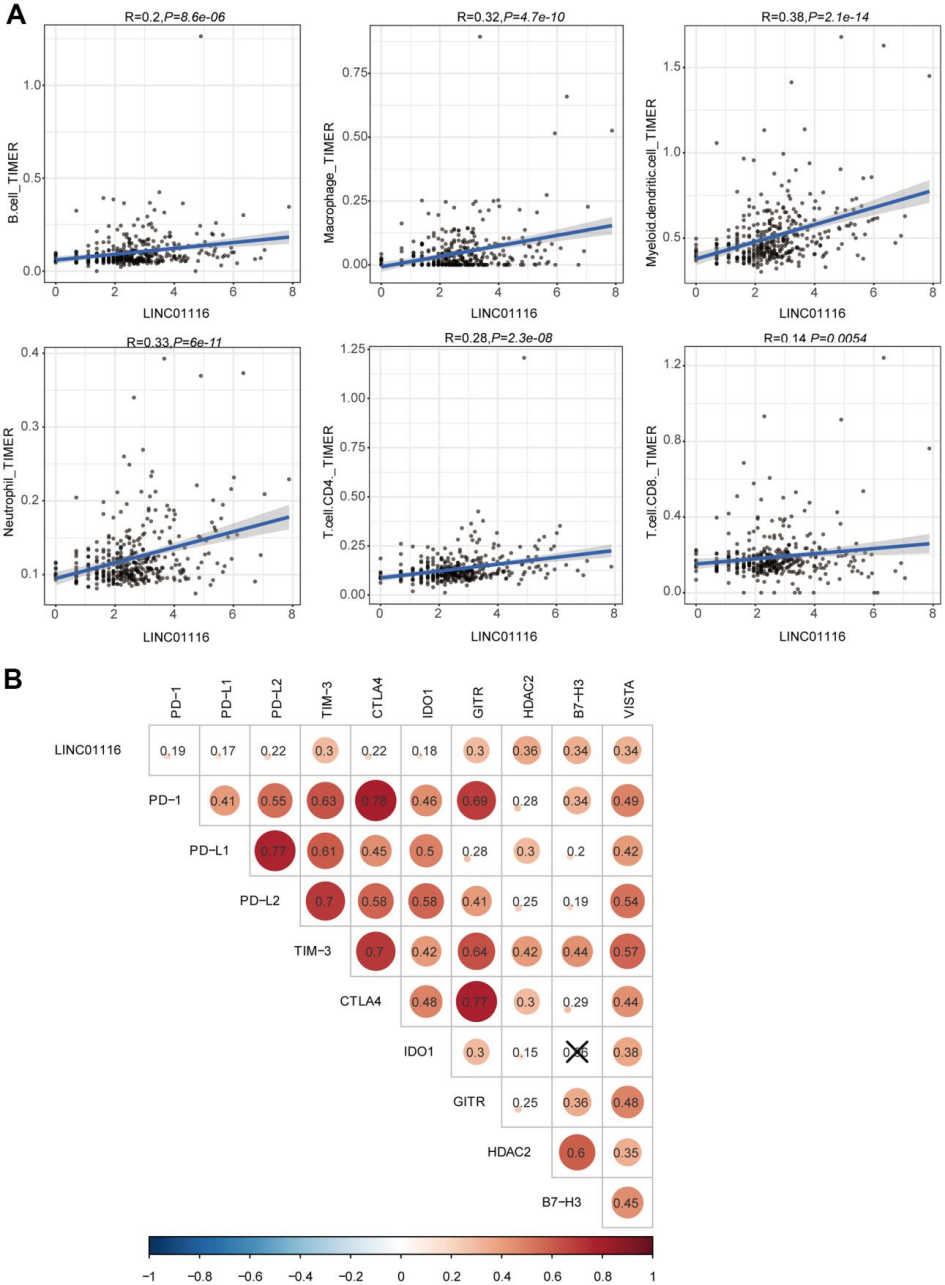
(**A**) Correlation analyses of LINC01116 with immune cell infiltration based on TIMER database. (**B**) Correlation analyses of LINC01116 with immune checkpoint targets.

As described above, LINC01116 expression was found to be significantly correlated with the infiltration of 6 immune cell types based on the CIBERSORT algorithm (Figure 6A). We further examined the relationship between LINC01116 and TIICs using TIMER. LINC01116 expression level was positively and significantly correlated with B cell, CD4+ T cell, CD8+ T cell, macrophage, neutrophil, and dendritic cell infiltration (Figure 10A). To evaluate the influence of LINC01116 on ICB therapy in HCC, we performed a correlation analysis between LINC01116 and immune checkpoint targets (CTLA4, TIM-3, PD-L1, PD-L2, PD-1, IDO1, GITR, HDAC2, B7-H3, and VISTA) and found positive correlations with the levels of all 10 targets (Figure 10B). Thus, LINC01116 plays a critical role in immune regulation in HCC and is a potential target for ICB therapy.

## DISCUSSION

EMT plays a critical role in cancer pathogenesis and metastasis [17]. Some lncRNAs are known to interact with and modulate the expression and functions of master regulators of EMT [18, 19]. Therefore, identifying EMT-related lncRNAs and elucidating their mechanisms of action in HCC can reveal novel therapeutic targets for precision medicine and thereby improve the prognosis of patients.

In the present study, EMT-related lncRNAs were screened and uni- and multivariate Cox and LASSO analyses were carried out to establish an EMT-related lncRNA signature. The prognostic value of the signature in HCC was evaluated by Kaplan–Meier, multivariate Cox, and ROC curve analyses. Patients with a high risk score were found to have more malignant HCC and significantly worse prognosis. The AUCs indicated that the signature could effectively predict the OS of HCC patients at 1, 3, and 5 years in different risk groups. Thus, the EMT-related lncRNA signature can guide risk stratification and clinical decision-making in HCC management.

EMT plays important roles in immune regulation and treatment response in cancer [20]. For example, tumor-associated macrophages and myeloid-derived suppressor cells in the tumor microenvironment induce EMT to facilitate immune evasion [21, 22]. EMT creates an immunosuppressive tumor microenvironment that contributes to low therapeutic response and resistance to anti–PD-L1 therapy [23]. Furthermore, some lncRNAs were shown to be involved in both immune evasion and EMT [24, 25]. Thus, EMT-related lncRNAs can potentially modulate the behavior of infiltrating immune cells and response to immunotherapy. This was supported by the fact that the EMT-related lncRNA signature established in our study was significantly associated with the degree of infiltration of multiple immune cell types. Although ICB therapy has achieved good results in various solid tumors, its efficacy in HCC is low [26, 27]. Enhancing the precision of ICB therapy in HCC by identifying more specific drug targets and biomarkers for predicting treatment response can improve clinical outcomes. The correlation analysis in the present work showed that the EMT-related lncRNA signature was significantly associated with all 10 immune checkpoint targets examined; moreover, the risk score of the signature increased with the expression levels of these targets. These results suggest that the EMT-related lncRNA signature can predict the responsiveness of HCC patients to ICB treatment.

Three hub lncRNAs (AC009005.1, AP002478.1, and LINC01116) in the EMT-related lncRNA signature are known to contribute to the malignant phenotype of various cancers, while AC025176.1 and AC011352.3 have not been previously described. AC009005.1 is an autophagy-related lncRNA that has been used to construct a prognostic model for HCC; it was shown to be more highly expressed in tumors than in normal liver tissue, which was associated with shorter OS of HCC patients [28]. AP002478.1 has also been incorporated into several prognostic models for HCC based on its upregulation in HCC tissues and association with poor outcome. Additionally, AP002478.1 was shown to be overexpressed in 4 HCC cell lines compared to the normal hepatocyte cell line LO2, suggesting an involvement in the development of HCC [29, 30]. LINC01116 has been linked to various cancers. LINC01116 activated interleukin (IL)-1β expression in glioma by regulating the transcriptional regulator DEAD-box helicase (DDX)5, thereby promoting neutrophil recruitment and glioma cell proliferation [31]. LINC01116 also modulated gefitinib resistance in non-small cell lung cancer (NSCLC) cells by regulating interferon-induced protein (IFI)44 expression, suggesting a potential mechanism for drug resistance in NSCLC [32]. Through competitive binding of the miRNA miR-31-5p, LINC01116 increased the expression of the proangiogenic factor vascular endothelial growth factor (VEGF) to promote glioma tumorigenesis [33].

All 5 hub lncRNAs in the EMT-related lncRNA signature were highly expressed in HCC tissues of the Tongji Hospital cohort, which is consistent with the TCGA data. Given that it had the highest expression in HCC specimens compared to nontumor tissue in the Tongji Hospital cohort and has not been previously characterized in HCC, LINC01116 was selected for more detailed study. We found that LINC01116 enhanced proliferation, cell cycle progression, and migration in HCC cells *in vitro*, indicating that it can promote EMT and metastasis in HCC. The results of *in vivo* experiments confirmed that overexpression of LINC01116 increased tumor cell proliferation and induced the expression of EMT-related factors. We also found that LINC01116 was related not only to immune cell infiltration but to the expression of 10 immune checkpoint targets. Taken together, our findings indicate that LINC01116 functions as an oncogene in HCC that participates in both EMT and immune regulation.

## CONCLUSIONS

In the present study we established an EMT-related lncRNA signature that can be used to predict the prognosis of HCC patients. We provided evidence that the signature is directly related to a high degree of malignancy in HCC and is closely associated with immune cell infiltration and multiple immune checkpoint targets. LINC01116, a hub gene in the signature, was identified as an immune-related oncogene in HCC and potential target for HCC immunotherapy. These findings can help to improve the management of HCC patients to achieve better clinical outcomes.

## Supplementary Material

Supplementary Figures
