# Supplementary material for: Identification of an EMT-related lncRNA signature and LINC01116 as an immune-related oncogene in hepatocellular carcinoma
Source: Aging (Albany NY). 2022 Feb 11;14(3):1473–91. doi: 10.18632/aging.203888 (PMC8876905; doi:10.18632/aging.203888)
Supplement: Supplementary Figures [file aging-14-203888-s001.pdf]

SUPPLEMENTARY FIGURES

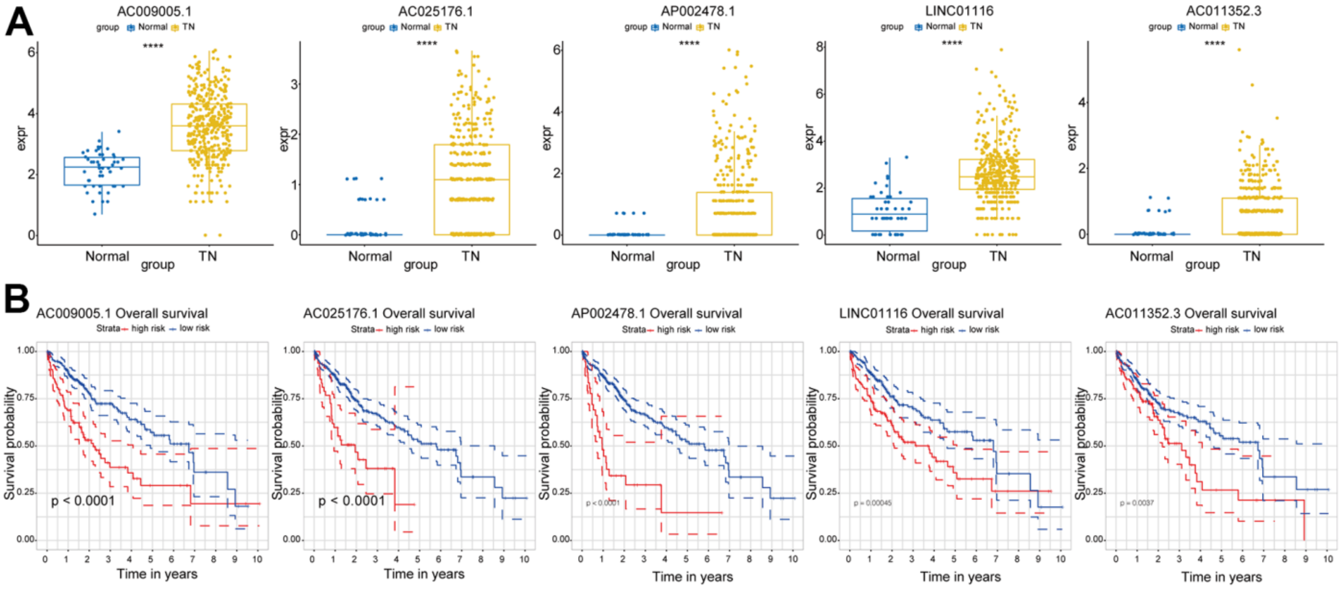

Supplementary Figure 1. (**A**) The expression levels of 5 hub lncRNAs in HCC specimens of TCGA. (**B**) Kaplan–Meier survival analysis of 5 hub lncRNAs.

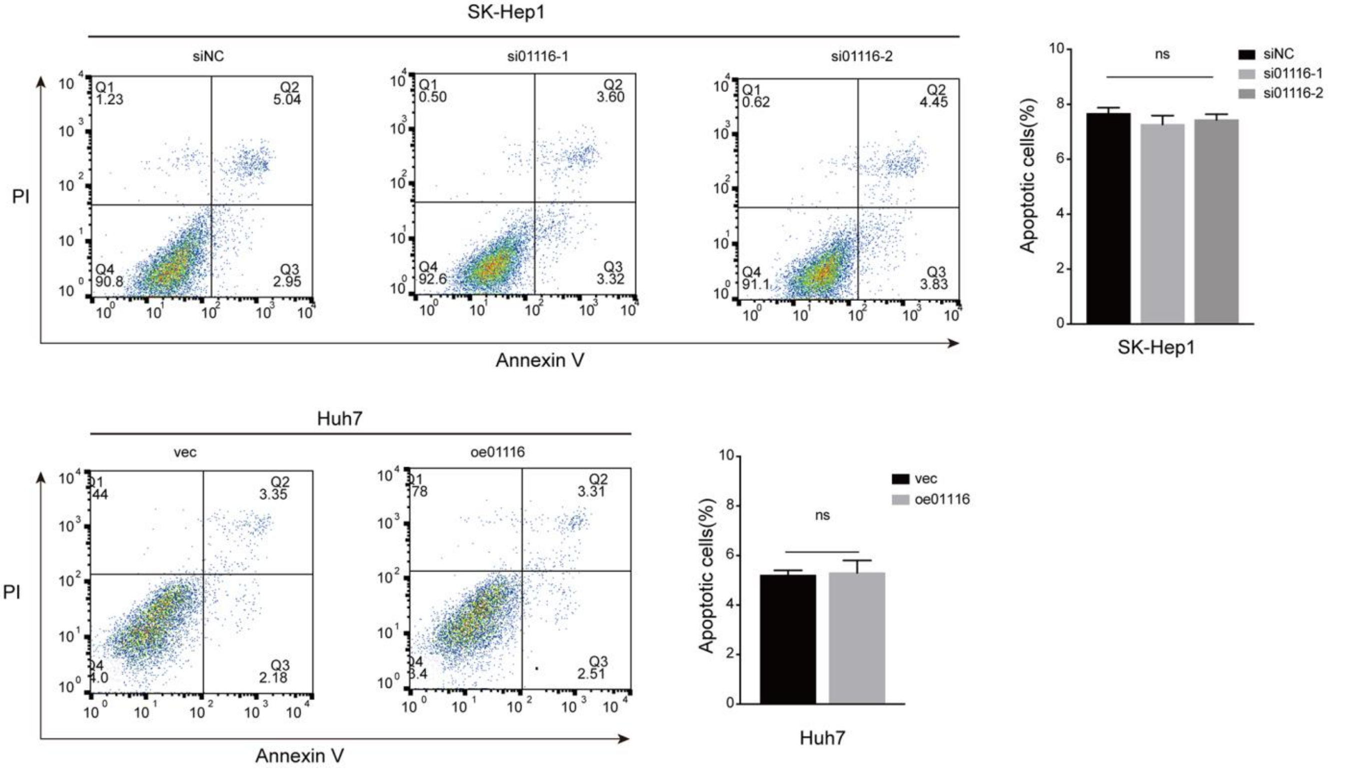

Supplementary Figure 2. Cell apoptosis was examined by the flow cytometry.
